# Conformational Targeting of Fibrillar Polyglutamine Proteins in Live Cells Escalates Aggregation and Cytotoxicity

**DOI:** 10.1371/journal.pone.0005727

**Published:** 2009-05-28

**Authors:** Erik Kvam, Brent L. Nannenga, Min S. Wang, Zongjian Jia, Michael R. Sierks, Anne Messer

**Affiliations:** 1 Wadsworth Center, New York State Department of Health, Albany, New York, United States of America; 2 Department of Biomedical Sciences, University at Albany, Albany, New York, United States of America; 3 Department of Chemical Engineering, Arizona State University, Tempe, Arizona, United States of America; National Institutes of Health, United States of America

## Abstract

**Background:**

Misfolding- and aggregation-prone proteins underlying Parkinson's, Huntington's and Machado-Joseph diseases, namely α-synuclein, huntingtin, and ataxin-3 respectively, adopt numerous intracellular conformations during pathogenesis, including globular intermediates and insoluble amyloid-like fibrils. Such conformational diversity has complicated research into amyloid-associated intracellular dysfunction and neurodegeneration. To this end, recombinant single-chain Fv antibodies (scFvs) are compelling molecular tools that can be selected against specific protein conformations, and expressed inside cells as intrabodies, for investigative and therapeutic purposes.

**Methodology/Principal Findings:**

Using atomic force microscopy (AFM) and live-cell fluorescence microscopy, we report that a human scFv selected against the fibrillar form of α-synuclein targets isomorphic conformations of misfolded polyglutamine proteins. When expressed in the cytoplasm of striatal cells, this conformation-specific intrabody co-localizes with intracellular aggregates of misfolded ataxin-3 and a pathological fragment of huntingtin, and enhances the aggregation propensity of both disease-linked polyglutamine proteins. Using this intrabody as a tool for modulating the kinetics of amyloidogenesis, we show that escalating aggregate formation of a pathologic huntingtin fragment is not cytoprotective in striatal cells, but rather heightens oxidative stress and cell death as detected by flow cytometry. Instead, cellular protection is achieved by suppressing aggregation using a previously described intrabody that binds to the amyloidogenic N-terminus of huntingtin. Analogous cytotoxic results are observed following conformational targeting of normal or polyglutamine-expanded human ataxin-3, which partially aggregate through non-polyglutamine domains.

**Conclusions/Significance:**

These findings validate that the rate of aggregation modulates polyglutamine-mediated intracellular dysfunction, and caution that molecules designed to specifically hasten aggregation may be detrimental as therapies for polyglutamine disorders. Moreover, our findings introduce a novel antibody-based tool that, as a consequence of its general specificity for fibrillar conformations and its ability to function intracellularly, offers broad research potential for a variety of human amyloid diseases.

## Introduction

Abnormal aggregation of polypeptides into amyloid-like fibrils is associated with more than 20 known human disorders collectively referred to as protein misfolding or conformational diseases [1]. Despite little shared sequence homology, amyloid-forming polypeptides show a common propensity to misfold into highly-ordered polymers that are rich in fibrillar β-sheet structure. Distinct amyloidogenic polypeptides are genetically implicated in the progression of human neurodegenerative disorders, including Alzheimer's, Parkinson's, polyglutamine, and prion diseases. Human polyglutamine disorders such as Machado-Joseph disease (MJD) and Huntington's disease (HD) are caused by aberrant codon expansion of CAG trinucleotide tracts within unrelated genes encoding polyglutamine-domain proteins. In HD, expansions beyond 37 consecutive glutamines within the huntingtin protein confer a toxic gain-of-function phenotype related to its intracellular aggregation in neurons [2]. Proteinaceous huntingtin aggregates are diagnostic hallmarks of HD neuropathology and coincide with neurological symptoms in humans [3] as well as in transgenic models of the disease [4]. These intracellular aggregates are composed chiefly of polyglutamine-containing amino-terminal fragments of huntingtin that arise by proteolysis. Consequently, the first exon of the human Huntingtin gene (Htt exon1) containing an expanded CAG repeat is sufficient to induce HD-like pathology, including intracellular aggregates and neurodegeneration, in transgenic mice models [5]. Likewise, neuronal protein inclusions are clinically observed in MJD and arise through aberrant expansion of the polyglutamine-encoding CAG repeat within the ataxin-3/MJD1/SCA3 locus [6].

Following destabilization of native protein folding by expanded polyglutamine domains, the aggregation of mutant huntingtin or ataxin-3 proceeds by nucleated growth polymerization into protein fibrils that structurally resemble amyloid fibrils and react with amyloid-specific histochemical dyes such as Congo Red or thioflavin [7], [8]. Analogous biophysical properties are observed for β-amyloid and α-synuclein fibrils associated with Alzheimer's and Parkinson's diseases, respectively [9]. Amyloid fibrils are thought to be nucleated by monomers or globular oligomers of misfolded protein [10]. In turn, fibrils may co-assemble into much larger insoluble protein aggregates that are resistant to proteolysis. While it is clear that protein misfolding can elicit cellular toxicity, whether fibrillar protein aggregates are themselves toxic remains the subject of intense debate. On one hand, a growing body of evidence supports the “toxic soluble precursor” hypothesis in which end-stage protein fibrils are increasingly considered benign or even cytoprotective [11], [12]. In support of this hypothesis, small molecules that visibly stimulate inclusion-formation inside cells appear to be beneficial [13]. However, contrasting studies have achieved compelling cytoprotection by preventing fibrils and aggregates from forming at all [14]. These competing hypotheses are not mutually exclusive given the growing diversity in “on-pathway” and “off-pathway” protein folding conformations observed during amyloidogenesis [15].

Conventional antibodies raised against amyloid conformations are known to cross-react with a wide variety of misfolded proteins, thereby illustrating that diverse amyloidogenic proteins share isomorphic features [16], [17]. However, these conformation-specific antibodies cannot be readily implemented inside living cells to investigate the conformational toxicity of intracellular amyloidogenic proteins such as huntingtin, α-synuclein, and ataxin-3 in situ. In an alternative approach, recombinant single-chain Fv (scFv) antibodies, which preserve the binding specificities of monoclonal antibodies within the framework of a single small polypeptide, can be selected in vitro and expressed intracellularly as “intrabodies” to probe huntingtin and α-synuclein in living cells [18], [19]. By encoding the antigen-binding site of an immunoglobulin within the framework of a single nucleic acid coding sequence, scFvs are amenable to genetic manipulation and expression as single genes [20]. A recently developed technique for screening recombinant scFv libraries against amyloidogenic protein morphologies has identified several human conformation-specific scFvs against oligomeric and fibrillar forms of α-synuclein [21], [22]. Here, we demonstrate that one such scFv antibody (scFv-6E) selected against fibrillar α-synuclein targets isomorphic conformations of mutant huntingtin and ataxin-3 and enhances the aggregation propensity of these polyglutamine proteins in striatal cells. Using intrabodies as kinetic tools for controlling amyloidogenesis, we show that accelerating polyglutamine aggregation is not cytoprotective but rather aggravates intracellular dysfunction and cell death. These findings validate the importance of aggregation kinetics in modulating the severity of polyglutamine-mediated neuronal dysfunction.

## Results

### Fibril-specific scFv-6E intrabody co-localizes with intracellular aggregates of misfolded httex1 and ataxin-3

**Figure 1:**
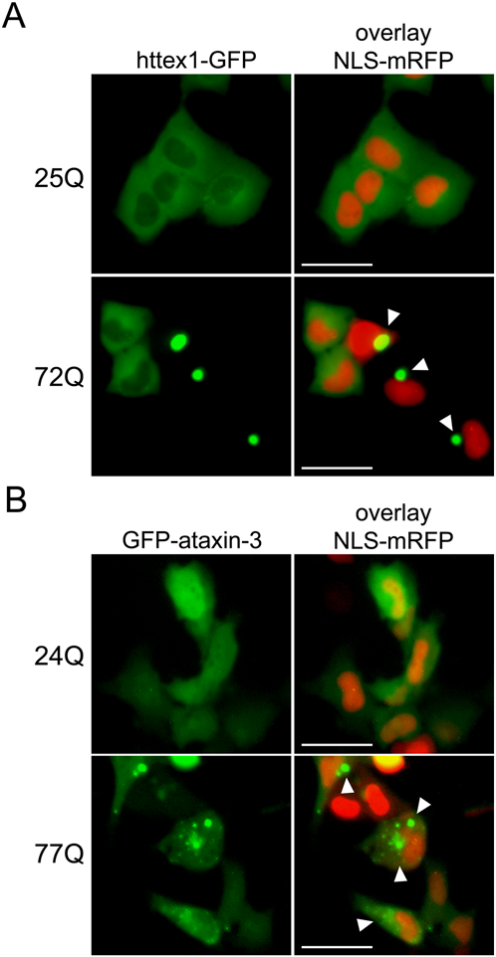
Localization of GFP-tagged httex1 and full-length ataxin-3 in live ST14A striatal progenitor cells. ST14A cells were transiently transfected with httex1-GFP or GFP-ataxin-3 reporters containing normal or disease-associated polyglutamine tract lengths, and NLS-mRFP to label nuclei. Representative live-cell images were captured 48 hours post-transfection as described in Materials and Methods. (A) GFP-tagged httex1-25Q localizes diffusely in the cytoplasm while mutant httex1-72Q precipitously forms large aggregates (arrows) in the cytoplasm, which often appear juxtaposed to nuclei. (B) GFP-tagged ataxin-3-24Q localizes diffusely within ST14A cells while mutant ataxin-3-77Q forms small aggregates (arrows) predominately in the cytoplasm. Scale bars, 25 µm.

Antibody scFv-6E was isolated by panning a synthetic human scFv library against purified α-synuclein amyloid fibrils using a combination of phage display and atomic force microscopy (AFM) techniques [22]. By ELISA, scFv-6E exhibits conformation-specific binding to fibrillar α-synuclein but not monomeric or oligomeric species [22]. Drawing from evidence that fibrils of diverse amyloidogenic proteins possess common structural epitopes [17], we reasoned that scFv-6E may function as a conformational sensor for other fibril-forming proteins such as polyglutamine proteins. Therefore, we tested scFv-6E in cellular models of polyglutamine disease using either human ataxin-3 (MJD1/SCA3) or a pathologic huntingtin fragment encoded by the first exon of the Huntingtin gene (Httex1), both of which form filamentous protein aggregates dependent on polyglutamine tract length [7], [8]. Through live-cell microscopy, we confirmed that green fluorescent protein (GFP)-tagged versions of these misfolding-prone proteins formed intracellular aggregates in immortalized rat striatal progenitor cells (ST14A) with similar characteristics as widely documented in the literature [23]–[26]. ST14A cells were chosen for this study because these neuronal cells share features with medium-spiny neurons [27], which are pre-disposed to HD neuropathology [28] and affected in models of MJD [29]. We included in our transfections a nuclear localization signal (NLS)-tagged monomeric red fluorescent protein (NLS-mRFP) to label nuclei. Aside from this general purpose, NLS-mRFP is a convenient marker for monitoring cellular stress in live cells, as its mislocalization to the cytoplasm is indicative of nuclear import collapse [30], [31]. As illustrated in Figure 1A, GFP-labeled mutant httex1, containing an expanded polyglutamine repeat (72Q) associated with juvenile HD onset, rapidly formed cytoplasmic and perinuclear aggregates in ST14A cells after 48 hours, whereas httex1 containing a non-amyloidogenic polyglutamine repeat (25Q) remained completely diffuse. Nuclear localization of httex1-GFP was rarely observed in ST14A cells, as reported in other culture models of HD [25], [26], [32]. The coalescence of misfolded httex1-72Q species into intensely fluorescent aggregates resulted in the disappearance of diffuse httex1-72Q-GFP species from the surrounding cytoplasm (Fig. 1A, arrows), highlighting the rapid precipitous nature of huntingtin aggregation [26]. Similarly, GFP-tagged mutant ataxin-3, containing an expanded polyglutamine tract (77Q) associated with MJD, predominately formed cytoplasmic aggregates in transfected ST14A cells over a similar time period whereas non-expanded ataxin-3 (24Q) localized diffusely (Fig. 1B). Unlike the case for httex1, ataxin-3 was not excluded from nucleoplasm and therefore nuclear aggregates of GFP-ataxin-3-77Q were occasionally present along with cytoplasmic aggregates. Striking differences in aggregate size and frequency were clearly evident between mutant ataxin-3 and httex1, as inclusions were observed in ∼70% of httex1-72Q transfected cells by 48 hours but in only ∼20% of ataxin-3-77Q transfected cells over a similar time period, with ataxin-3-77Q inclusions appearing much smaller than httex1 inclusions. These observations likely reflect the fact that ataxin-3 was expressed in its native full-length form while httex1 represents an N-terminal fragment of the huntingtin protein that is generated by proteolysis [33].

**Figure 2:**
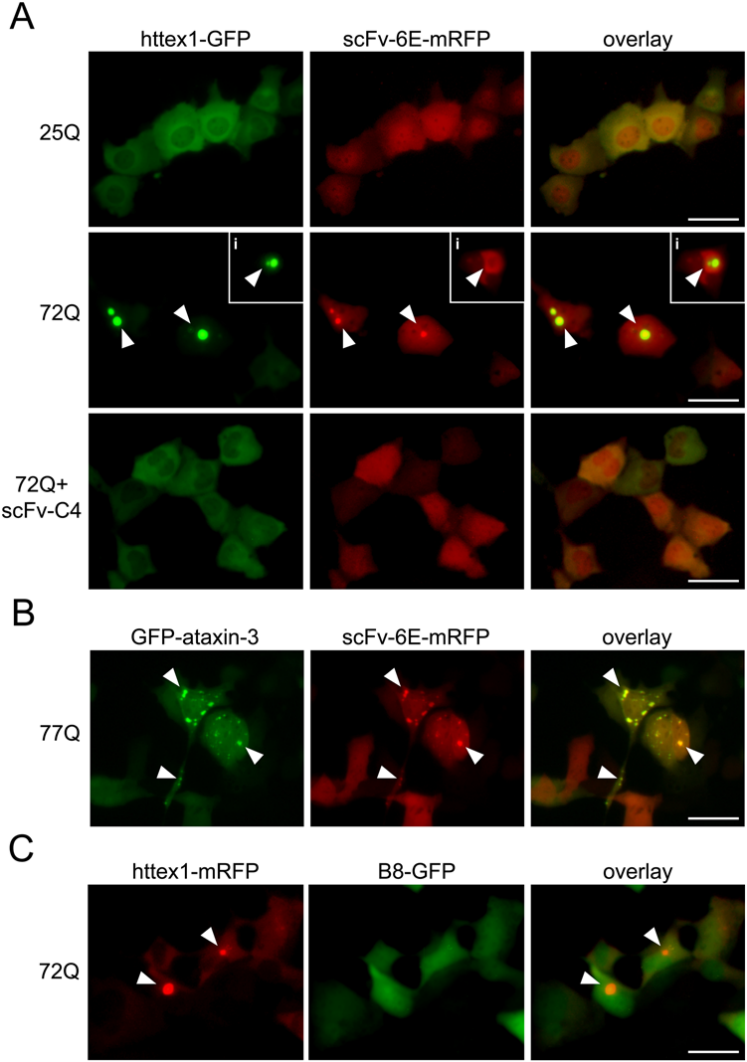
scFv-6E fluorobodies co-localize with intracellular aggregates of mutant httex1 and ataxin-3 in live cells. ST14A cells were transiently transfected with the indicated GFP- and mRFP-tagged reporters, and representative live-cell images were captured 48 hours post-transfection as described in Materials and Methods. (A) mRFP-tagged scFv-6E fluorobody co-localizes as intracellular puncta with cytoplasmic aggregates of GFP-tagged httex1-72Q (arrows), occasionally in a ring-like pattern (inset i). Transfection with unlabeled scFv-C4 efficiently suppresses co-localization of scFv-6E fluorobody with inclusions by inhibiting the aggregation of httex1-72Q-GFP. In the absence of httex1 inclusions, scFv-6E fluorobody exhibits a diffuse localization pattern. (B) mRFP-tagged scFv-6E fluorobody co-localizes with cytoplasmic and nuclear aggregates of GFP-tagged ataxin-3-77Q (arrows). (C) GFP-labeled control intrabody (B8) selected against botulinum neurotoxin light chain protease fails to co-localize with mutant httex1-72Q-mRFP inclusions. Scale bars, 25 µm.

To determine whether scFv-6E antibody co-localizes with such filamentous polyglutamine inclusions, we fused scFv-6E to the amino-terminus of enhanced monomeric red fluorescent protein (mRFP Q66T) to create traceable scFv-6E “fluorobodies” that can be directly visualized in live cells through fluorescence microscopy [34]. To minimize folding interference, a flexible (Gly_4_Ser)_4_ peptide linker was cloned between scFv-6E and mRFP to ensure that each moiety folds independently and produces a functional fluorescent scFv fusion, as suggested in other studies [35]–[37]. When co-expressed with fluorescently-labeled httex1 or ataxin-3 possessing disease-related polyglutamine repeats, we observed that scFv-6E fluorobodies formed intracellular puncta that co-localized with aggregates of mutant httex1-72Q and ataxin-3-77Q (Fig. 2A, B). In some cells, scFv-6E fluorobodies encircled polyglutamine inclusions in a ring-like pattern (Fig. 2A, inset i), a feature reminiscent of the recruitment of specific binding partners onto the surface of protein aggregates [38]. In contrast, scFv-6E fluorobody adopted a diffuse localization pattern in the presence of non-amyloidogenic httex1-25Q-GFP, and showed little specific overlap with soluble httex1-25Q species as indicated by clear differences in the nuclear versus cytoplasmic localization of these two reporters (Fig. 2A). Importantly, co-localization of scFv-6E fluorobody with httex1-72Q inclusions could be suppressed by blocking aggregation using a second intrabody, scFv-C4, that binds within the first 17 amino-terminal residues of huntingtin [19] (Fig. 2A). In the presence of scFv-C4, fibril-specific scFv-6E fluorobody shifted from a punctate localization to a diffuse pattern (Fig. 2A), suggesting that scFv-C4 intrabody blocked the formation of misfolded httex1 epitopes normally recognized by scFv-6E. As an additional control, GFP-labeled scFv-C4 fluorobody completely inhibited httex1-72Q aggregation in live ST14A cells and co-localized perfectly with non-aggregated httex1-72Q in the cytoplasm (Fig. S1), demonstrating that our fluorobody design generates functional fluorescently-tagged intrabodies. Moreover, a nonspecific control fluorobody (B8-GFP) selected against botulinum neurotoxin light chain protease [39] failed to co-localize with aggregates of fluorescently-labeled httex1-72Q in ST14A cells (Fig. 2C), suggesting that the observed co-localization of scFv-6E fluorobody with polyglutamine inclusions is direct. In total, these co-localization experiments support our hypothesis that scFv-6E is a conformation-specific antibody fragment capable of distinguishing disease-related amyloid proteins with filamentous structure inside cells.

### 6E scFv binds httex1 amyloid fibrils and enhances fibrillogenesis in solution

**Figure 3:**
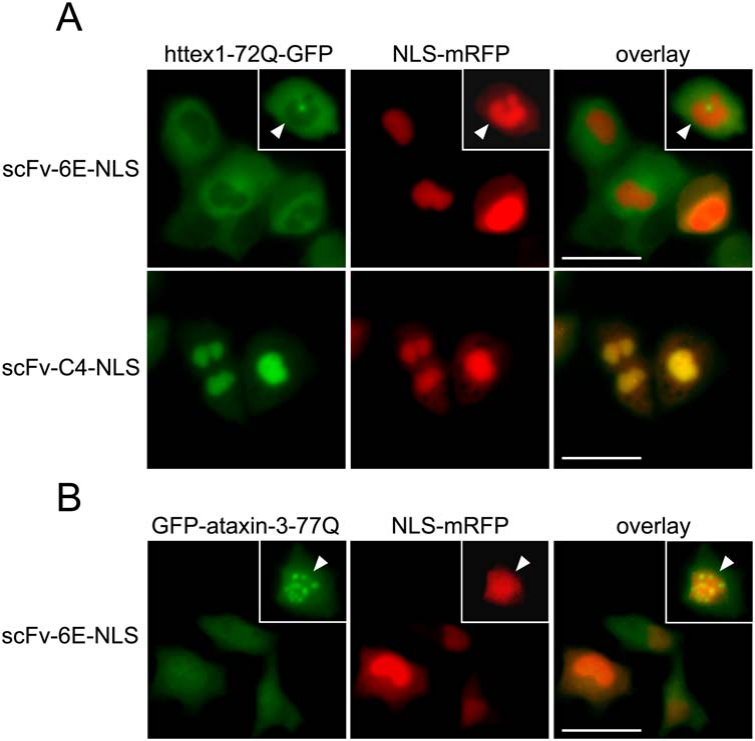
NLS-fused scFv-6E intrabody retargets polyglutamine aggregates but not soluble polyglutamine species in situ. Antibody-antigen interactions were probed in ST14A cells using NLS-fused scFv-6E to sequester both intrabody and bound antigen into nuclei. NLS-mRFP fluorescence marks nuclei. Representative live-cell images were captured as described in Materials and Methods. (A) NLS-fused scFv-6E sequesters small cytoplasmic aggregates of GFP-labeled httex1-72Q (inset, arrow) but not soluble species into nuclei. In contrast, a control NLS-fused intrabody (scFv-C4-NLS) completely recruits soluble httex1-Q72-GFP species into nuclei from the cytoplasm. (B) NLS-fused scFv-6E completely sequesters aggregates of GFP-labeled ataxin-3-77Q (inset, arrow) but not soluble species into nuclei. Scale bars, 25 µm.

To confirm that scFv-6E binds fibrillar huntingtin species directly, we employed an AFM-based in vitro technique for measuring antibody affinity to specific protein morphologies [40]. Following standard protocols for generating httex1 fibrils containing an expanded polyglutamine tract (51Q) typical of adult HD onset [8], mutant httex1 fibrils were deposited onto freshly cleaved mica for surface height analyses by AFM. By measuring changes in the surface height of fibrils before and after incubation with scFv antibodies, we were able to detect that purified 6E scFv, but not a control scFv against phosphorylase B (αPLB), bound specifically to fibrillar httex1-51Q (Table 1). Binding of scFv-6E to httex1-51Q fibrils increased the average surface height of these fibrils by nearly 2-fold compared to identical fibrils incubated with a control scFv (αPLB) or in buffer alone (Table 1). Similar increases in particle height were observed when mature α-synuclein fibrils, but not purified oligomeric or monomeric α-synuclein species, were incubated with scFv-6E in vitro [40]. These results support prior conclusions that misfolded huntingtin and α-synuclein exhibit common structural amyloid epitopes, as first revealed by others using conformation-specific conventional antibodies [16], [41]. To confirm our AFM findings and test whether scFv-6E may also be specific for soluble conformers of mutant protein, we next assayed for direct antibody-antigen interactions in ST14A cells using subcellular re-localization assays. A potent nuclear localization sequence (NLS) derived from SV40 Large T antigen was fused to scFv-6E to target intrabody/antigen complexes into nuclei. By transient transfection, we observed that scFv-6E-NLS failed to recruit soluble species of either mutant httex1-72Q-GFP or GFP-ataxin-3-77Q into nuclei (Fig. 3A, B), contrary to a control NLS-tagged intrabody (scFv-C4-NLS, Fig. 3A) shown previously to bind and sequester soluble httex1 species into nuclei [19]. Instead, we observed that scFv-6E-NLS intrabody sequestered tiny cytoplasmic aggregates of both GFP-ataxin-3-77Q and httex1-72Q-GFP into nuclei (Fig. 3A, B, insets), although larger aggregates often remained perinuclear likely due to steric constraints at nuclear pores. In total, these findings validate our fibril specificity data in Table 1 and demonstrate that scFv-6E is specific for a conformational epitope typical of insoluble amyloid-like aggregates but not soluble species of mutant protein.

**Table 1:** Mean particle height of httex1-51Q fibrils incubated with/without scFvs.

Treatment	n	Mean height (nm)^a^	SD (nm)	p-value
buffer	52	2.51	±0.53	NA
6E scFv	53	4.25	±0.90	<0.0001
αPLB scFv	48	2.47	±0.56	0.7438

a Surface height measurements were obtained by cross-sectional analysis of httex1-51Q fibrils deposited on mica and incubated with or without scFvs as described in Materials and Methods. Absolute maximum z-heights were averaged from 10–20 cross-sections per fibril using line profiles drawn perpendicular to the fibril axis. Fibrils (n) were analyzed in three separate sections of mica per sample. P-values represent statistical significance from buffer-treated control sample. SD, standard deviation; nm, nanometers; αPLB, anti-phosphorylase B.

**Figure 4:**
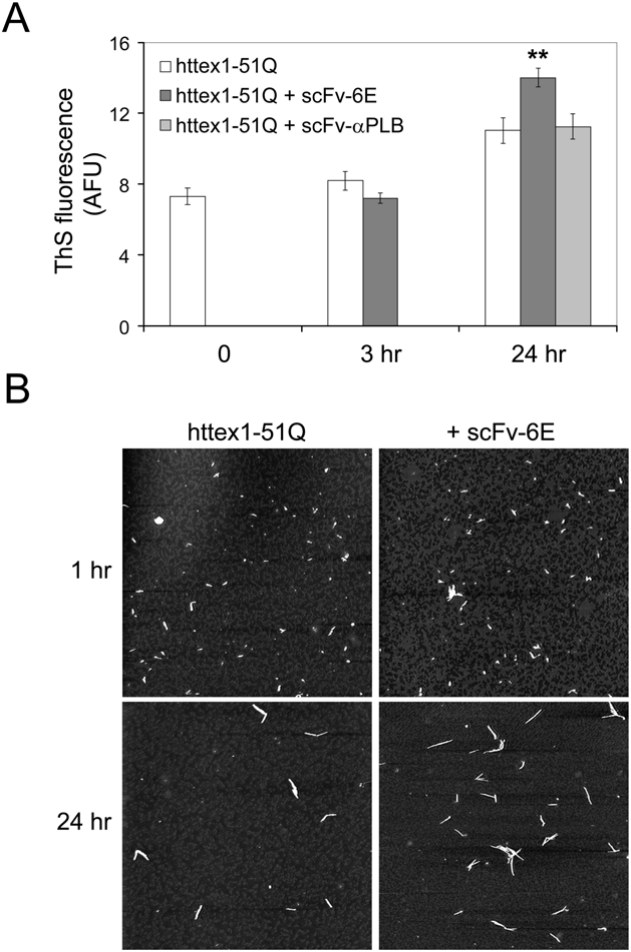
scFv-6E enhances the fibrillar aggregation of mutant httex1 in solution. (A) Fluorescence intensity of Thioflavine S-stained httex1-51Q fibrils formed in the presence or absence of scFv antibodies. Purified httex1-51Q, scFv-6E, and scFv-αPLB proteins were prepared as described in Materials and Methods. Samples were analyzed 0, 3, and 24 hours after initiating aggregation. Each bar represents mean Thioflavine S fluorescence (arbitrary fluorescence units, AFU) from reactions performed in triplicate. Statistical significance (** p<0.01) was determined by ANOVA. (B) Representative AFM images of httex1-51Q aggregation reactions conducted in the absence or presence of scFv-6E. AFM scan size, 5 µm×5 µm.

Given that scFv-6E binds fibrils, we next investigated whether scFv-6E altered mutant httex1 aggregation kinetics in solution. Cell-free, de novo aggregation studies of purified httex1-51Q protein were conducted in the absence or presence of scFv-6E, and fibril-formation was monitored spectrophotometrically using Thioflavine S (ThS), an amyloid-specific dye that undergoes a spectral shift upon binding fibrils [1]. Approximately 24 hours after initiating aggregation, httex1-51Q samples incubated with scFv-6E exhibited 27±5% higher levels of ThS fluorescence on average than buffer-treated control samples, thus indicating enhanced fibrillar aggregation (Fig. 4A). No increases in ThS fluorescence were observed at earlier time points during httex1-51Q oligomerization (Fig. 4A) or after 24 hour incubation with a control scFv against phosphorylase B (Fig. 4A). AFM imaging of these samples confirmed that httex1-51Q fibrils were more abundant following incubation with fibril-specific scFv-6E (Fig. 4B). Importantly, httex1-51Q fibrils appeared structurally identical between samples incubated with or without scFv-6E (Fig. 4B) and displayed branched morphologies characteristic of mutant huntingtin [42]. To validate the binding specificity of scFv-6E, protein aliquots of mutant httex1 from pre-cleared control reactions were applied onto nitrocellulose at specific time points during fibrillogenesis and probed against scFv-6E by dot-blot analysis. These experiments confirmed that scFv-6E binds httex1 at time points when fibrils predominate (24, 144 hours) but not at earlier time points (0, 1, 6 hours) when monomers and oligomers prevail (Table 2). In total, these in vitro findings imply that scFv-6E likely enhances the kinetic aggregation of httex1-51Q by binding and stabilizing nascent fibrils, thereby reducing the thermodynamic lag-time of fibrillogenesis.

**Table 2:** Relative staining intensity of scFv-6E probed by dot-blot against mutant httex1 at specific time points during fibrillogenesis^a^.

Time point	0	1 hour	6 hours	24 hours	144 hours
scFv-6E staining intensity	1.00	1.10±0.28	1.28±0.14	2.22±0.20	3.32±0.18

a Staining intensities were determined by dot-blot analysis of httex1-53Q using scFv-6E as a primary antibody. Briefly, purified GST-httex1-53Q protein was ultracentrifuged to remove preformed particles and digested to initiate httex1-53Q aggregation as described in Materials and Methods. Aliquots of httex1-53Q were deposited onto nitrocellulose 0, 1, 6, 24, and 144 hours after initiating aggregation and probed against scFv-6E by dot-blot. Results from duplicate experiments were averaged and normalized to time 0 values. Higher scFv-6E staining is observed at later time points when httex1-53Q is predominately fibrillar but not earlier time points when monomers and oligomers prevail.

### scFv-6E intrabody enhances the intracellular aggregation of mutant httex1

**Figure 5:**
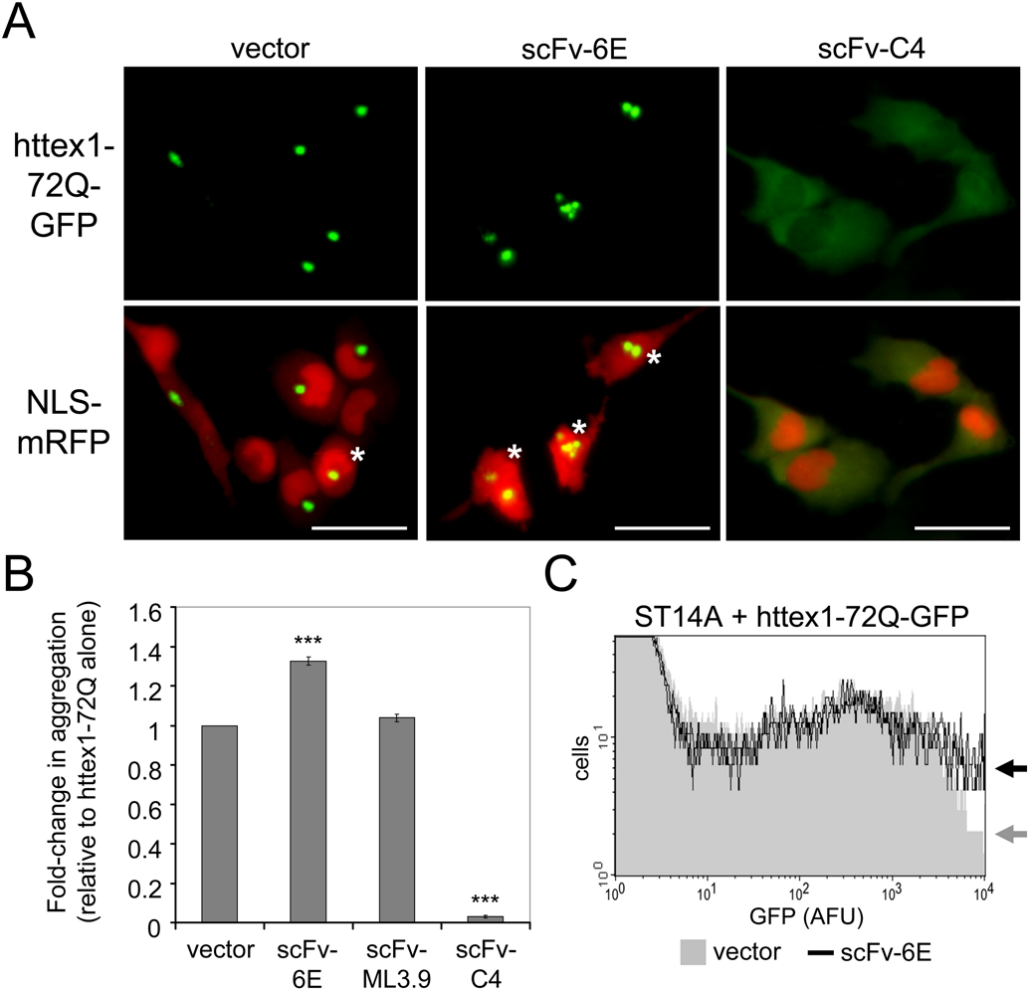
scFv-6E intrabody enhances mutant httex1 aggregation in ST14A cells. (A) Representative live-cell images depicting the localization patterns of httex1-72Q-GFP and NLS-mRFP in the absence or presence of intrabodies. scFv-6E intrabody increases the frequency of cytoplasmic httex1-72Q aggregates while scFv-C4 intrabody blocks aggregate formation in ST14A cells. Asterisks denote cells where NLS-mRFP nuclear import is impaired, indicating cell stress. Scale bars, 25 µm. (B) Relative aggregation propensity of httex1-72Q in the absence or presence of intrabodies. Aggregates of httex1-72Q-GFP were scored as described in Materials and Methods. Statistical significance (*** p<0.0001) was determined by ANOVA (n = 4). (C) FACS histogram of httex1-72Q-GFP fluorescence intensity (arbitrary fluorescence units, AFU) plotted as a function of cell number. ST14A cells co-expressing httex1-72Q-GFP and either scFv-6E (black) or empty vector (gray) were sorted by fluorescence intensity as described in Materials and Methods. Representative data from triplicate experiments were superimposed.

We next tested if scFv-6E enhanced the aggregation of mutant httex1 when expressed as an intrabody inside neuronal cells. To this end, unlabeled scFv-6E was cloned downstream of a strong viral promoter and co-expressed with mutant httex1-72Q-GFP in ST14A cells. At 48 hours post-transfection, we observed 33±2.2% more GFP-labeled httex1-72Q aggregates in the cytoplasm of cells co-transfected with scFv-6E intrabody than with vector alone (Fig. 5A, B), in good agreement with our in vitro findings (Fig. 4). Due to the stochastic nature of httex1 aggregation, aggregate frequency increased both population-wide and per cell following expression of scFv-6E, with qualitatively more cells containing multiple cytoplasmic aggregates (Fig. 5A). Interestingly, NLS-mRFP was generally mislocalized to the cytoplasm of aggregate-laden cells (Fig. 5A, asterisks), indicating a collapse in nuclear import, which is a hallmark of cellular stress [30], [31]. By exploiting the fact that the fluorescence intensity of httex1-72Q-GFP increases during aggregation (Fig. 1A), we were able to confirm by flow cytometry that scFv-6E augments httex1-72Q aggregation in situ. Cells with intense httex1-72Q-GFP fluorescence (10^3^–10^4^ arbitrary fluorescence units, AFU) increased in frequency following expression of scFv-6E intrabody compared to vector alone (Fig. 5C, arrows), indicating a proliferation in GFP-labeled mutant httex1 inclusions. Importantly, no significant differences in GFP fluorescence were observed at lower peak intensities (10^1^–10^3^ AFU), thereby ruling out the possibility that httex1 expression differences could account for this result. Taken together, we conclude that scFv-6E enhances mutant httex1 aggregation in situ as well as in solution. These findings starkly contrasted with experiments conducted in parallel using anti-huntingtin scFv-C4 intrabody, which strikingly blocked mutant httex1 aggregation in ST14A striatal cells (Fig. 5A, B), consistent with published observations in other models [19], [43].

**Figure 6:**
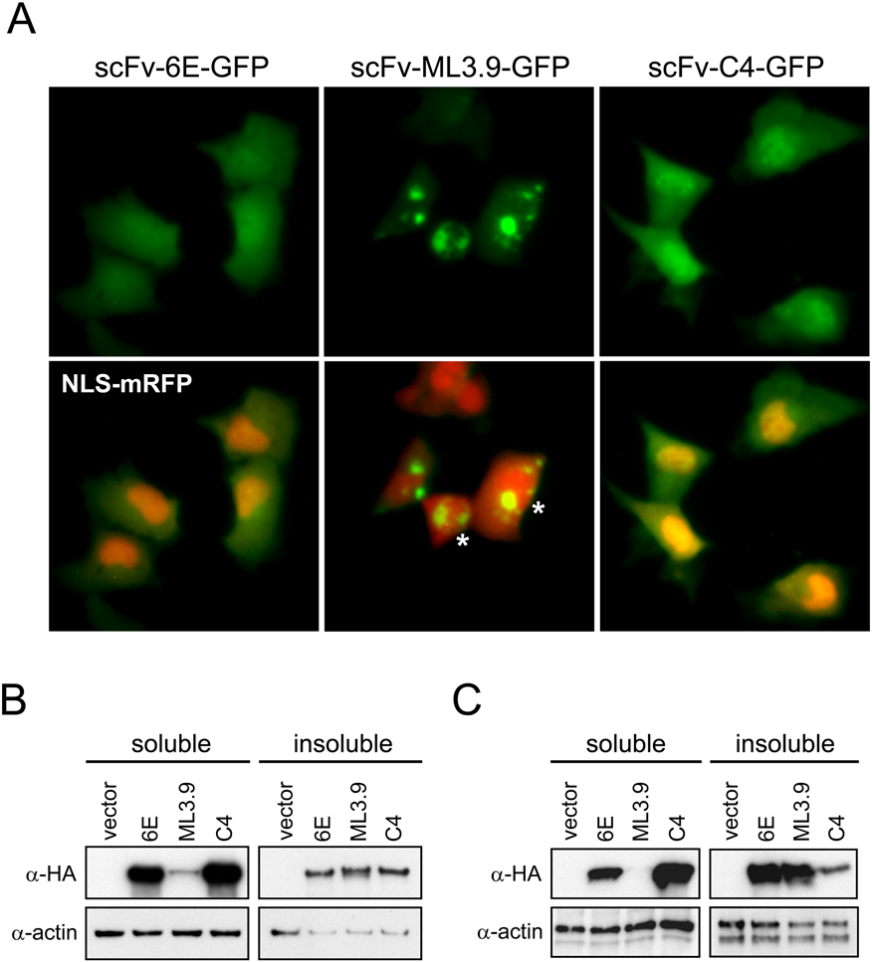
Relative intrabody solubility. (A) Live images of ST14A cells co-transfected with GFP-tagged fluorobodies and NLS-mRFP, a nuclear marker. Cells were imaged 48 hours post-transfection as described in Materials and Methods. scFv-6E fluorobody predominately localizes as a diffuse soluble protein similar to anti-huntingtin scFv-C4 fluorobody. scFv-ML3.9 fluorobody localizes almost entirely as inclusions. Note NLS-mRFP nuclear import is perturbed in the presence of scFv-ML3.9 inclusions (asterisks), indicating cell stress. (B) Biochemical fractionation of HA-tagged fluorobodies from ST14A cells. Detergent-soluble and -insoluble cell lysates were prepared as described in Materials and Methods. (C) Biochemical fraction of HA-tagged intrabodies following elevated overexpression. Detergent-soluble and -insoluble cell lysates were prepared as described in Materials and Methods.

Recombinant scFv antibodies are generally destabilized when expressed as cytoplasmic intrabodies due to the redox-inhibition of intrachain disulfide bonds that normally play a role in antibody folding [44]. We recognized this fact could directly influence the aggregation kinetics of huntingtin, since studies have shown that destabilized proteins may function as non-specific modifiers of polyglutamine misfolding by sequestering chaperones and eliciting global protein folding stress [45]. To test if the observed effects of scFv-6E intrabody on httex1 aggregation kinetics might be indirect, we repeated our intracellular studies using a nonspecific scFv (ML3.9) against the extracellular domain of the cell surface tumor receptor c-erbB-2 [46]. When expressed in cell cytoplasm as a GFP-labeled fluorobody, scFv-ML3.9 exhibited striking insolubility as indicated by the formation of intracellular aggregates, in contrast to scFv-6E and -C4 fluorobodies, which generally exhibited diffuse cytoplasmic localization patterns consistent with soluble proteins (Fig. 6A,B). Despite such intrinsic instability in cell cytoplasm, scFv-ML3.9 intrabody failed to significantly enhance the intracellular aggregation of httex-72Q-GFP in ST14A cells as judged by our scoring assay (Fig. 5B), consistent with prior observations in other cell types [19]. By analogy, we conclude that the stimulatory effects of scFv-6E intrabody on httex1 aggregation are direct, and cannot be simply explained by folding stress related to intrabody expression. This conclusion is further supported by the fact that scFv-6E localizes and generally fractionates as a soluble intracellular protein (Fig. 6A, B), even when expressed at elevated levels to increase the potential for folding stress (Fig. 6C), as well as the fact that scFv-6E directly enhances fibrillar aggregation of mutant httex1 in cell-free experiments (Fig. 4).

### scFv-6E and -C4 intrabodies differentially affect httex1-dependent toxicity in situ

**Figure 7:**
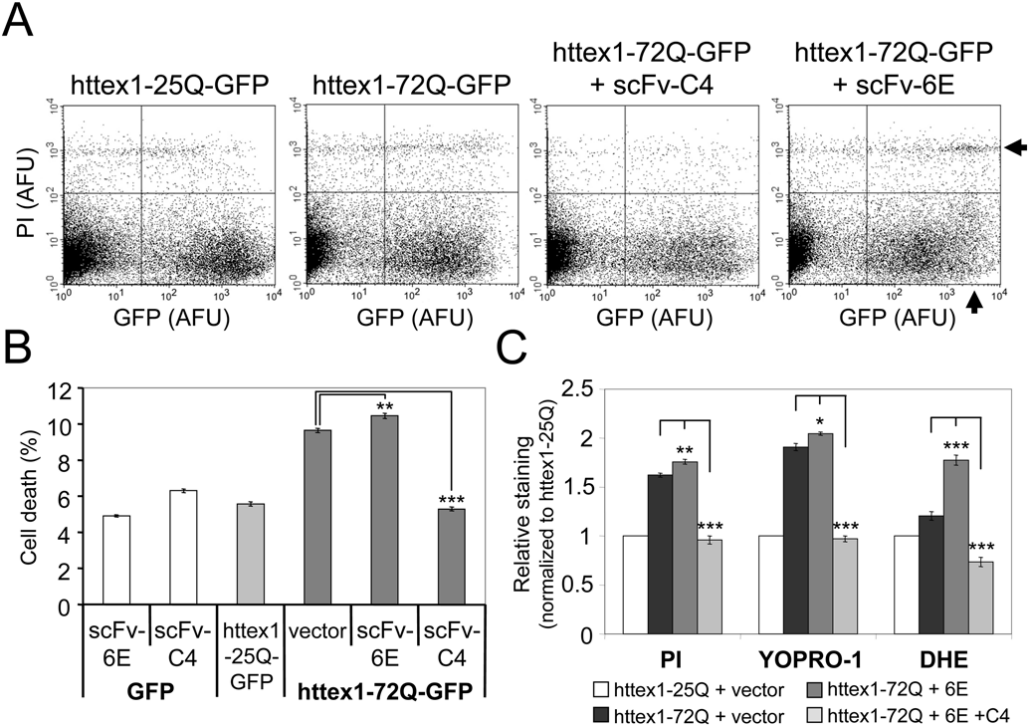
scFv-6E and -C4 intrabodies differentially affect mutant httex1-mediated toxicity and oxidative stress in situ. (A) Two-color FACS dot-plots from representative experiments in which ST14A cells were transiently transfected with the indicated reporters, stained with propidium iodide (PI) to label dead cells, and co-sorted for GFP and PI fluorescence intensity. A total of 30,000 cells were plotted on a logarithmic scale of arbitrary fluorescence intensity. Grid lines were positioned after calibrating the flow cytometer as described in Materials and Methods. Lower right quadrants demarcate viable transfected cells (GFP^+^ PI^−^). Upper right quadrants signify PI-labeled dead or dying transfected cells (GFP^+^ PI^+^). Note that scFv-6E intrabody shifts httex1-72Q-GFP fluorescence to a higher intensity among PI-labeled cells (arrows), indicating that these cells died with intensely fluorescent aggregates. (B) Percentage of cell death observed by two-color FACS analysis with PI. Cell death was calculated by dividing the number of PI-stained transfected cells (GFP^+^ PI^+^, see upper right quadrants in A) over the total number of transfected cells (GFP^+^ PI^+^ and GFP^+^ PI^−^, see upper and lower right quadrants in A). Statistical significance from httex1-72Q-GFP alone (** p<0.001, *** p<0.0001) was determined by ANOVA (n = 3). (C) Relative cell staining of PI, YOPRO-1, and dihydroethidium (DHE) dyes as detected by two-color FACS. ST14A cells were transiently transfected with the indicated GFP- or mRFP-labeled httex1 reporters (with or without scFv-6E and scFv-C4 intrabodies) and incubated with PI, YOPRO-1, or DHE as described in Materials and Methods. Cell staining percentages were calculated by dividing the number of dye-stained transfected cells by the total number of transfected cells, and data were normalized to non-expanded httex1-25Q alone. Statistical significance from expanded httex1-72Q alone (* p<0.01, ** p<0.001, *** p<0.0001) was determined by ANOVA (n = 3).

A growing body of evidence has suggested that protein inclusions large enough to be visible by light microscopy might be irrelevant to misfolded-protein pathology or even cytoprotective [47]. The ability of scFv-6E to target intracellular amyloids offered a unique opportunity to test these models in situ. Using huntingtin as a paradigm, we compared the viability of ST14A cells expressing GFP-labeled mutant httex1 in the presence or absence intrabody. Approximately 48 hours post-transfection, ST14A cells were incubated with propidium iodide (PI), a membrane-impermeant dye that labels necrotic cell nuclei following membrane damage, and PI fluorescence was co-sorted against GFP-labeled httex1 fluorescence by flow cytometry. As shown in Figure 7A–C, expression of mutant httex1-72Q-GFP alone resulted in greater PI staining and toxicity than non-amyloidogenic httex1-25Q-GFP. Accelerating the aggregation of mutant httex1 with scFv-6E intrabody elicited a further increase in PI-staining (Fig. 7B, C), particularly among cells exhibiting large or numerous inclusions as indicated by a prominent forward shift in GFP fluorescence intensity (10^3^–10^4^ AFU, Fig. 7A, see arrows) within these cells. This small but statistically significant (p<0.001) increase in cell death is consistent with the modest increase in httex1-72Q aggregate load (33±2.2%) elicited by scFv-6E (Fig. 5A, B). In contrast, blocking httex1-72Q-GFP aggregation with scFv-C4 intrabody completely abolished polyglutamine-dependent toxicity (Fig. 7A, B). More importantly, scFv-C4 intrabody also suppressed mutant httex1 aggregation and toxicity in the presence of scFv-6E intrabody (Fig. 7C, 2A). These results confirm that scFv-C4 functions upstream of scFv-6E with regard to mutant httex1 aggregation kinetics. As an additional control for specificity, cells expressing intrabody in the presence of GFP alone showed virtually no toxicity (Fig. 7B).

To verify our findings, we repeated the above experiments using YOPRO-1, a DNA-intercalating dye that labels apoptotic as well as necrotic cells [48]. Because YOPRO-1 emits fluorescence in the green spectra, we cloned and expressed mRFP-labeled httex1 reporters that emit red fluorescence. By flow cytometry, we observed a pattern of YOPRO-1 staining that exactly paralleled our PI results, as the cellular toxicity of mutant httex1-72Q increased in an aggregation-specific manner with scFv-6E intrabody but was completely suppressed upon blocking aggregation with scFv-C4 intrabody (Fig. 7C). Thus, our findings illustrate that escalating mutant httex1 aggregate formation is not cytoprotective, but rather enhances polyglutamine-dependent toxicity. However, we should stress that total cell death remained relatively low (<15%) among cells expressing mutant httex1-72Q with or without scFv-6E intrabody (Fig. 7B), and thus the vast majority of cells bearing httex1 aggregates were overwhelmingly viable within this 48 hr period. Taken together, we conclude that the aggregation of mutant httex1 is not instantly toxic to ST14A striatal-derived cells but rather gradually compromises viability over time.

### scFv-6E intrabody augments httex1-mediated oxidative stress

Recent studies have highlighted a role for huntingtin in eliciting mitochondrial dysfunction and oxidative stress [49], [50]. We observed by live-cell fluorescence microscopy that cells with mutant httex1 inclusions tended to exhibit defects in NLS-mRFP nuclear import (Fig. 5A, see asterisks), which can be a consequence of oxidative stress [30], [31]. To directly test if the aggregation of mutant httex1 in fact exacerbates oxidative stress, we incubated transiently transfected ST14A cells with dihydroethidium (DHE, also known as hydroethidine), a fluorescent redox indicator that is readily cell-permeant. As a consequence of elevated oxidative stress and reduced mitochondrial membrane potential, DHE becomes enzymatically oxidized, in part, to ethidium bromide, which binds DNA and emits a bright red fluorescence [51]. Using flow cytometry, we quantified the production of ethidium bromide in DHE-stained ST14A cells expressing GFP-tagged httex1 and either intrabody or vector. As shown in Figure 7C, oxidation of DHE increased concomitantly with httex1-72Q aggregation in the presence of fibril-specific scFv-6E intrabody but was completely suppressed by blocking aggregation with scFv-C4 intrabody. Fluorescence microscopy confirmed that ST14A cells possessing httex1-72Q aggregates showed increased ethidium bromide fluorescence after loading with DHE, consistent with prior studies [50], [52]. In total, these data imply that escalating the intracellular aggregation of httex1 is cytotoxic to ST14A striatal-derived cells due, in part, to increased oxidative stress.

### scFv-6E intrabody boosts human ataxin-3 aggregation and toxicity

**Figure 8:**
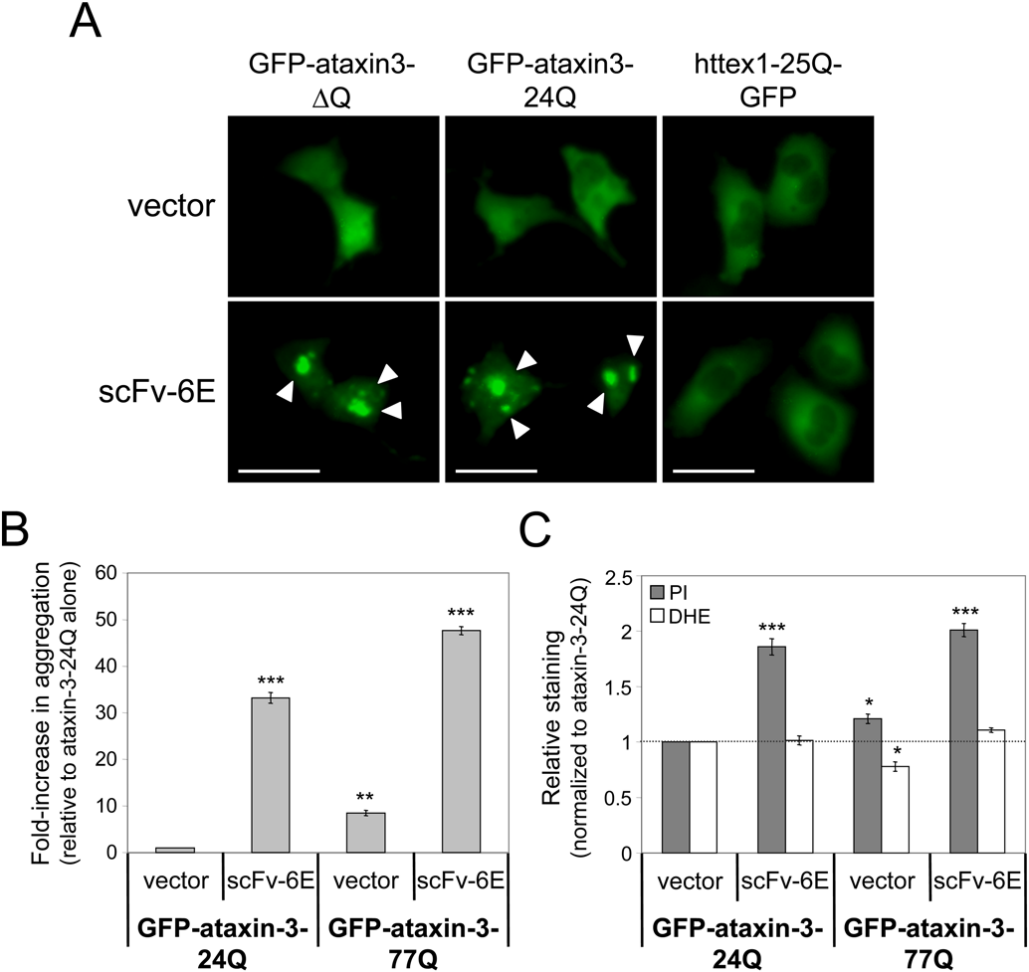
scFv-6E intrabody enhances aggregation and toxicity of both normal and disease-associated ataxin-3. (A) Live-cell images depicting GFP localization patterns of non-disease-associated ataxin3-24Q, ataxin3-ΔQ (lacking a polyglutamine tract), or httex1-25Q in the absence or presence of scFv-6E intrabody. scFv-6E intrabody stimulates the intracellular aggregation of ataxin-3 (arrows) but not httex1-25Q. Scale bars, 25 µm. (B) Relative aggregation propensity of normal (24Q) or disease-linked (77Q) ataxin-3 in the absence or presence of scFv-6E intrabody. GFP-tagged ataxin-3 aggregates were scored in ST14A as described in Materials and Methods. Statistical significance (** p<0.001, *** p<0.0001) was determined by ANOVA (n = 3). (C) Relative cell staining of propidium iodide (PI) and dihydroethidium (DHE) vital dyes as detected by two-color FACS. ST14A cells were transiently transfected with normal (24Q) or disease-linked (77Q) GFP-ataxin-3 and either scFv-6E intrabody or empty vector. Cell staining percentages were calculated as described in Materials and Methods, and data were normalized to GFP-ataxin-3-24Q alone. Statistical significance (* p<0.01, *** p<0.0001) from GFP-ataxin-3-24Q was determined by ANOVA (n = 3).

To extend our analysis and confirm that scFv-6E accelerates protein amyloidogenesis, we tested scFv-6E against full-length human ataxin-3, which possesses a unique propensity to aggregate through non-polyglutamine domains [53]. Thus, unlike the case for huntingtin, polyglutamine expansion is not required for nucleating ataxin-3 fibrillogenesis in solution [53]. Rather, self-interactions mediated by the amino-terminal Josephin domain of ataxin-3 are thought to initiate fibrillogenesis [7], [54]. However, in vitro studies demonstrate that ataxin-3 fibrils formed in the absence of long polyglutamine tracts are less stable than those formed by expanded ataxin-3; thus, expanded polyglutamine plays a modulating role in the thermodynamics and kinetics of ataxin-3 fibrillogenesis [7], [53]. Given that scFv-6E antibody binds fibrils and appears to reduce the lag-time of huntingtin fibrillogenesis (Figs. 4, 5), we hypothesized that scFv-6E may also accelerate the aggregation of ataxin-3 via conformational stability. Hence, we transfected ST14A cells with GFP-labeled versions of non-expanded (24Q) or polyglutamine-deleted (ΔQ) ataxin-3, and tested for accelerated ataxin-3 inclusion formation in the presence of scFv-6E intrabody by live-cell fluorescence microscopy. As shown in Figure 8A, non-expanded GFP-ataxin-3 (24Q) exhibited a mostly diffuse and non-aggregated localization pattern alone but formed multiple intracellular aggregates in the presence of scFv-6E intrabody after 48 hours. Similar results were observed with GFP-labeled ataxin-3 deleted of polyglutamine (ΔQ) (Fig. 8A). In contrast, scFv-6E intrabody failed to stimulate the visible aggregation of non-expanded httex1 (25Q) (Fig. 8A), which is not known to form fibrils naturally [42]. Together, these results demonstrate that fibril-specific scFv-6E kinetically escalates intracellular amyloidogenesis through a conformation-specific and thermodynamic mechanism that is independent of protein sequence.

We next investigated the relationship between ataxin-3 aggregation and toxicity using a combination of microscopy and flow cytometry. GFP-labeled aggregates of pathologic (77Q) or non-disease-associated (24Q) ataxin-3 were scored in ST14A cells in the presence or absence of intrabody, and cellular toxicity and oxidative stress were measured using PI and DHE vital dyes, respectively. These analyses revealed that ataxin-3 toxicity increased concomitantly with aggregation propensity (Fig. 8B, C), even when ataxin-3 contained a non-disease-associated polyglutamine repeat (24Q). Thus, non-pathologic ataxin-3 could be transformed into a toxic protein by stabilizing and accelerating its intrinsic ability to aggregate using scFv-6E. Compared to parallel data collected against mutant httex1 (Fig. 5B, 7C), these 6E-mediated effects appeared more robust with ataxin-3, likely due to kinetic differences in the amyloidogenic properties of these two polyglutamine proteins. In the absence of intrabody, we noted that ataxin-3 formed fewer aggregates than mutant httex1 in ST14A cells, suggesting that the amyloidogenesis of full-length ataxin-3 occurs with a longer lag-phase inside cells, and thus a greater window of opportunity may exist for scFv-6E intrabody to influence aggregation. Interestingly, oxidative stress was not significantly associated with ataxin-3 aggregation and toxicity as measured using DHE (Fig. 8C), unlike the case for mutant httex1 (Fig. 7C). These findings validate that the downstream toxic effects of amyloidogenesis are highly dependent on protein sequence, and support the notion that sequences other than the polyglutamine domains of httex1 and ataxin-3 influence the etiology of cellular dysfunction through distinct protein-interaction networks [55]. Thus, conformation-specific intrabodies such as scFv-6E offer broad research potential for investigating the biogenesis and consequence of protein misfolding and aggregation across a wide-spectrum of human amyloid disorders.

## Discussion

Intracellular protein aggregation is linked to the onset and progression of a variety of human amyloid disorders, although whether or not visible protein inclusions are innately toxic remains a matter of contention. Accumulating evidence from HD and other amyloidogenic disease models has led to the “toxic soluble precursor” hypothesis, which proposes that toxic intermediates formed during amyloidogenesis might be “detoxified” through their polymerization into insoluble amyloid-like fibrils [11]. As a result, visible protein aggregates are increasingly thought to fulfill a protective cellular response. This model is indirectly supported by cytoprotective evidence obtained using small molecules that promote the intracellular aggregation of huntingtin as well as α-synuclein [13] via an undetermined mechanism. In this study, we demonstrate a direct enhancement of huntingtin and ataxin-3 aggregation using a fibril-specific scFv antibody (scFv-6E) that discriminates intracellular aggregates in situ. This conformation-specific scFv recognizes a fibrillar epitope also characteristic of α-synuclein, which scFv-6E was originally selected against [22]. Since little sequence homology exists between α-synuclein, huntingtin, and ataxin-3, we speculate that scFv-6E recognizes a structural epitope common to amyloid morphologies such as the cross β-sheet motif. As a result, conformation-specific scFv antibodies such as 6E have broad research potential for a variety of human amyloid disorders. Using scFv-6E as a kinetic tool for enhancing amyloidogenesis, we show that targeting aggregation of mutant huntingtin in striatal cells is not protective, but rather promotes oxidative stress and cell death. These results are consistent with prior findings demonstrating a role for misfolded huntingtin in eliciting mitochondrial dysfunction and oxidative stress [49], [50]. A similar inter-relationship between aggregation and toxicity is observed for both normal and disease-associated ataxin-3, although through a mechanism apparently distinct from oxidative stress. In total, these results lend caution to the pursuit of therapeutic strategies aimed at enhancing protein aggregation as treatments for HD and related polyglutamine disorders [47]. Interestingly, preliminary cellular studies using scFv-6E intrabody to target fibrillar α-synuclein suggest that a different therapeutic strategy may apply for synucleinopathies compared to polyglutamine diseases (S. Lynch, unpublished data).

The data presented in this report are consistent with numerous studies supporting an aggregation model for polyglutamine pathogenesis. Elevated toxicity is reported in several HD models after stimulating aggregation through such means as chaperone depletion [56], [57], dopamine exposure [58], inhibiting autophagy [59], expressing anti-polyglutamine recombinant intrabodies [60], depleting normal cellular prion protein [61], or overexpressing specific huntingtin-interacting proteins such as intersectin or normal repeat-length huntingtin fragments [62], [63]. Our findings argue against a protective role of protein inclusions in sequestering and detoxifying soluble misfolded intermediates, since accelerating the rate of fibrillogenesis would likely decrease the concentration of such “on-pathway” intermediate conformers. Instead, we observed that cellular toxicity ultimately correlated with the formation of visible polyglutamine aggregates. Consistent with this observation, blocking aggregate-formation using an intrabody (scFv-C4) that binds within the first 17 amino acid residues of huntingtin potently suppressed httex1-mediated toxicity, analogous to its protective role in other HD models [19], [43], [64]. We demonstrated previously that scFv-C4 intrabody binds preferentially to non-aggregated NH_2_-terminal huntingtin fragments and disrupts aggregation de novo, thereby eliciting greater turnover of httex1 fragments in situ [65]. Recent data now show that the epitope of scFv-C4 (aa 1–17 of huntingtin) is a membrane-binding amphipathic helix sufficient for targeting mitochondrial and ER membranes and necessary for triggering the aggregation of expanded huntingtin fragments [49], [66], [67]. These properties may explain how scFv-C4 intrabody mechanistically reduces aggregation, oxidative stress, and toxicity, namely by shielding this amyloidogenic huntingtin domain.

Several important factors likely explain why our results disagree with the findings of Bodner et al [13]. This group identified a cytoprotective compound called B2 that indiscriminately boosts the size of httex1 and α-synuclein inclusions, perhaps through an inhibitory effect on SIRT2 histone deacetylase [68]. Since compound B2 does not interact directly with huntingtin or α-synuclein, its cytoprotective effects may be pleiotropic and unrelated to inclusion body formation, analogous to the protective role of heat-shock protein Hsp27, which shows indirect activity in modulating huntingtin aggregation but a more fundamental role in antagonizing caspase activation and apoptosis in HD models [69]. In contrast, our fibril-specific scFv-6E antibody directly stimulates httex1 aggregation in solution and co-localizes with ataxin-3 and httex1 inclusions in situ, suggesting that scFv-6E interacts directly with amyloidogenic polypeptides through a conformational epitope produced during fibrillogenesis. It is possible that scFv-6E intrabody, by increasing aggregate load, might expand the protein-recruiting surface area of intracellular aggregates, which in turn could enhance toxicity by sequestering more aggregate-interacting proteins away from their native functions. In this context, compound B2 appears to decrease the net surface area of aggregates by amassing multiple small aggregates into a single large inclusion [13]. This apparent relationship between aggregate size and “toxic surface area” deserves further investigation, especially with regard to intracellular amyloidogenic proteins in compound aggregated states such as inclusion bodies and aggresomes. Alternatively, we cannot exclude the possibility that scFv-6E may stabilize a recently described oligomeric species having fibrillar features [16], although the existence of such oligomers has yet to be correlated with cytopathology. On this subject, it is noteworthy that the fibrillogenesis of ataxin-3 is initiated by a monomeric nucleus and grows by monomer addition rather than through oligomers in solution [7], and a similar mechanism may hold true for mutant httex1 as well [70]–[72].

Paradoxically, viable cells containing intracellular aggregates significantly outnumbered those that died with aggregates in our cytotoxicity experiments, demonstrating that aggregates per se are not instantly toxic to ST14A striatal-derived cells. This observation is consistent with previous reports demonstrating a penchant for inclusion bodies to be protective [12], [73], a fact which has called into question how visible protein aggregation can be associated with neuropathological abnormalities even though aggregates themselves are not universally predictive of neurodegeneration [3], [74]. Using intrabodies to alter the kinetics of amyloidogenesis, our data illustrates that the overall speed of aggregate formation bears more relevance to the severity of intracellular dysfunction than whether or not inclusions are present at any given time point. Indeed, time-lapse microscopy in a PC12 cell model of HD has revealed that aggregation tends to occur rapidly in cells that die with inclusions and much slower in cells that survive with inclusions [73]. We conclude that the rate of aggregation likely influences the magnitude of cellular dysfunction elicited by aggregation, such as oxidative stress in the case of mutant httex1. Hence, escalating the formation of httex1 aggregates with a fibril-specific intrabody heightens oxidative stress and results in greater toxicity in ST14A striatal cells. In contrast, suppressing aggregation with an intrabody that shields the amyloidogenic N-terminus of mutant huntingtin in fact reduces oxidative stress and toxicity in these cells. Importantly, oxidative stress complements several prevailing models of HD pathogenesis, including excitoxicity, mitochondrial dysfunction, and inflammation, as evidenced by the ability of the mitochondrial poison 3-nitropropionic acid (3-NP) to mimic striatum-specific HD-like motor defects [75]. Conversely, ataxin-3 is implicated in the regulation of endoplasmic reticulum (ER)-associated degradation [76]; thus, it is tempting to speculate that stimulating the aggregation of ataxin-3 might exacerbate ER stress-related dysfunction. In total, our findings support the general notion that polyglutamine aggregate-formation contributes to neurodegeneration by sensitizing cells to physiologic or environmental stress. In this regard, blocking or slowing aggregation elicits a strong cytoprotective effect in our study. These implications may hold importance for other protein misfolding diseases such as Amyotrophic Lateral Sclerosis (ALS), tauopathies, and Parkinson's, Alzheimer's, and prion disorders.

## Materials and Methods

### Expression plasmids

cDNA encoding scFv-6E (GenBank accession number FJ695518) was amplified by PCR using complementary primers that introduced a 5′ Kozak initiation sequence for mammalian expression and a 3′ HA epitope (YPYDVPDYA) for biochemical detection. The resulting PCR product was ligated downstream of the strong CMV promoter in pAAV-MCS (Stratagene) using standard cloning techniques. C4 (GenBank accession number EU490426) and ML3.9 [46] scFvs were cloned into pAAV-MCS in a similar manner. To create fluorescently-labeled intrabodies, humanized enhanced green fluorescent protein (EGFP) was PCR-amplified (without a start codon) using a complementary forward primer that introduced an N-terminal (Gly_4_Ser)_4_ flexible peptide repeat. This flexible peptide linker normally mediates proper folding of V_H_ and V_L_ antibody domains in scFv frameworks but has been adapted by others to create functional scFv-GFP fusions [35], [36]. PCR-generated (Gly_4_Ser)_4_-EGFP fragments were cloned into the 3′ end of the multiple cloning site of pcDNA3.1(-) (Invitrogen) to accommodate N-terminal fusion with intrabodies. Next, cDNAs of HA-tagged 6E, ML3.9, and C4 scFvs or untagged B8 [39] were amplified by PCR (without stop codons) and ligated upstream of and in-frame to (Gly_4_Ser)_4_-EGFP, resulting in the following orientation: Kozak sequence-intrabody-HA-(Gly_4_Ser)_4_-EGFP-stop. 6E intrabody was tagged with mRFP by replacing EGFP in the above vector with improved monomeric red-fluorescent protein (mRFP1 Q66T) [77]. Vectors for the expression of GFP-labeled human httex1 with different polyglutamine repeat lengths (pcDNA3.1-Httex1-25Q-EGFP, pcDNA3.1-Httex1-72Q-EGFP) were described elsewhere [78]. Httex1 was labeled with mRFP by replacing EGFP in the above vectors with mRFP1 (Q66T) [77] using standard cloning techniques. Likewise, mRFP (Q66T) was N-terminally tagged with an SV40 NLS (MTPPKKKRKV) using PCR primers and ligated into pcDNA3.1(-) (Invitrogen) to create NLS-mRFP1. Through a similar manner, SV40 NLS (TPPKKKRKV) was cloned onto the C-terminus of scFv-6E or scFv-C4 in pAAV-MCS (Stratagene) to target these intrabodies into nuclei. Vectors for the expression of GFP-labeled full-length human ataxin-3 with different polyglutamine repeat lengths (pEGFP-C1-Ataxin3-24Q; pEGFP-C1-Ataxin3-Q77) were described elsewhere [24]. A version of ataxin-3 lacking its polyglutamine domain [7] was PCR-amplified from pQE30-Ataxin-3 (QHQ) and subcloned into pEGFP-C1 (Clontech) using standard cloning techniques. All expression plasmids were confirmed by DNA sequencing.

### Cell culture and transfection

Undifferentiated ST14A cells derived from embryonic day 14 rat striatal primordia were propagated at 33°C essentially as described [27]. Endotoxin-free plasmid DNA was prepared using EndoFree Plasmid Maxi (Qiagen) and transiently transfected into ST14A cells using jetPEI DNA transfection reagent (Polyplus-transfection Inc.) at an N/P ratio of 8. Total amounts of transfected plasmid were reduced to 60% of the manufacturer's recommended amount and culture medium was replaced ∼3 hours post-transfection to minimize cellular toxicity. For all transfections, intrabody plasmids were applied at equal (1∶1) or lesser ratios to httex1 and ataxin-3 plasmids, and cells were analyzed 48 hours post-transfection.

### Cell-free protein aggregation assays

6E and α-PLB scFv antibodies were purified over protein A-Sepharose as previously described [22]. Recombinant httex1-51Q protein fused to glutathione S-transferase (GST-Httex1-51Q) was purified from *E. coli* and trypsinized to remove GST and initiate aggregation as described [79]. Surface height analyses of mature httex1-51Q fibrils were conducted similar to described [40]. Briefly, mature httex1-51Q fibrils were deposited on freshly cleaved mica and incubated at room temperature for 10 minutes. Fibril-coated mica were rinsed three times with distilled water and dried under a gentle stream of N_2_ gas. For scFv binding, purified 6E or α-PLB scFvs were incubated on fibril-coated mica for an additional 10 minutes followed by copious rinsing and drying under N_2_ gas. Fibril height measurements were performed at three different sections of mica using Scanning Probe Imaging Processor (SPIP) software (Image Metrology). Approximately 10 to 20 cross-sectional surface heights were measured per fibril using line profiles drawn perpendicular to the fibril axis. Absolute maximum Z-heights were recorded from each line profile and averaged among each sample.

For in vitro kinetic aggregation experiments, purified httex1-51Q was incubated at 37°C with either buffer or scFv-6E at equimolar ratios in triplicate. Samples were collected and analyzed at 0, 1, 3, and 24 hours after mixing. Fibril-formation was quantified using a 25 µM solution of Thioflavine S (Sigma) in PBS (pH 7.4). 25 µL aliquots of each aggregation reaction (with or without scFv-6E) were added to 2 mL of ThS solution, and fluorescence intensity was measured on a Shimadzu RF-5301 spectrofluorophotometer (430 nm excitation, 510 nm emission). The background fluorescence of ThS alone was subtracted from each measurement, and triplicate data were averaged. In parallel, 10 µL sample aliquots were deposited on cleaved mica and imaged by AFM similar to described [21]. Topographic images were recorded at a resolution of 512 samples per line.

For dot-blot analysis of scFv-6E specificity, freeze-dried GST-httex1-53Q protein was dissolved in pH 8.0 Tris solution containing 150 mM NaCl, 0.1 mM EDTA, and 2% glycerol, and subjected to ultracentrifugation (50,000 g×18 hours, 4°C) to remove any preformed particles. Pre-cleared GST-httex1-53Q protein (0.1 µg/µl) was digested with PreScission Protease (GE Healthcare) to remove GST and initiate aggregation. Aliquots of httex1-53Q were incubated at room temperature and 2.5 µg total protein was deposited onto nitrocellulose at 0, 1, 6, 24, and 144 hours after initiating aggregation. Dot-blots were air-dried after applying protein and then incubated with purified scFv-6E, labeled with anti-myc and HRP-conjugated anti-mouse antibodies to immunoprobe scFv-6E, and developed using DAB colorimetric methods. Staining intensities from duplicate experiments were quantified and normalized using ImageJ software (http://rsb.info.nih.gov/ij/).

### Cell fractionation and Western blot

Transiently transfected ST14A cells were collected from 6-well culture dishes by trypsinization and washed twice with PBS. Whole-cell lysates were extracted at 4°C in 50 µL/well of RIPA lysis buffer (50 mM Tris pH 7.5, 150 mM NaCl, 1% NP40, 0.25% sodium deoxycholate, 0.1% SDS) supplemented with 1× Complete protease inhibitor cocktail (Roche). Detergent-insoluble material was pelleted by microcentrifugation (13,000 rpm×10 minutes, 4°C). Soluble protein in the supernatant was quantified using Bio-Rad D_C_ Protein Assay kit (Bio-Rad Laboratories). Lysates were adjusted to 2 µg/µL in 2× denaturing sample buffer (125 mM Tris, 4% SDS, 20% glycerol, 10% 2-mercaptoethanol, 0.02% bromophenol blue, pH 6.8) and boiled. Insoluble pellet fractions were washed with RIPA, resuspended in 50 µL/well of 2× denaturing sample buffer, and boiled for 15 minutes. Equal volumes of pellet and soluble fractions corresponding to ∼20 µg of total protein (10 µL) were resolved by SDS-PAGE and transferred onto PVDF membranes (PerkinElmer). HA-tagged intrabodies and endogenous actin were probed with monoclonal α-HA (1∶1000, Covance) and α-actin (1∶1000, Sigma) Abs, respectively, labeled with HRP-conjugated goat-anti-mouse Ab (1∶2000, Santa Cruz), and detected by ECL (PerkinElmer).

### Live-cell imaging and aggregation counts

ST14A cells transfected with GFP- and/or mRFP-tagged reporters were imaged directly in 6-well culture dishes using an Olympus IX70 inverted microscope equipped with an Olympus IX-FLA Inverted Reflected Light Fluorescence Observation attachment and RGB Mirror Cube filter wheel (Olympus). To maximize detection of the fluorescent signal, culture medium was replaced with sterile PBS immediately prior to imaging, and cells were observed without fixation using a 40× lens. Images were captured 48 hours post-transfection with a SPOT RT Color CCD camera using SPOT Advanced software (Diagnostic Instruments). Camera exposure times were reduced when imaging protein aggregates due to their intense fluorescence. Digital images were overlayed and cropped using Adobe Photoshop. GFP-labeled httex1 or ataxin-3 aggregates were scored in live ST14A cells by counting the number of visual fluorescent aggregates from 5 separate 20×-objective fields, each showing high percentages of transfected cells, in a single well. The summations of these counts (number of observed aggregates per well) were averaged among several independent transfections, and data were normalized to ST14A cells expressing httex1-72Q-GFP or GFP-ataxin-3-24Q without intrabody, respectively.

### Flow cytometry and toxicity assays

ST14A cells were seeded into 6-well culture dishes (2×10^5^ cells/well) and transfected ∼24 hours later, in triplicate wells, with plasmids encoding GFP- or mRFP-tagged httex1 (containing either normal or expanded polyglutamine repeat tracts) and either intrabody or empty vector. Approximately 48 hours post-transfection, cell medium was harvested and adherent cells were collected by trypsinization (typically 6–8×10^5^ total cells/well). Harvested cells and exhausted media were combined and passed through a 70 µm cell strainer (BD Biosciences). Cells were pelleted by centrifugation (4,000 rpm×5 minutes) and resuspended in 1 mL of FACS buffer (1× Ca^2+^- and Mg^2+^-free PBS, 10% FBS) containing 50 µM propidium iodide (Sigma) or 0.5 µM YOPRO-1 (Invitrogen). Cells were stained for 20 minutes in the dark and pelleted by centrifugation (4,000 rpm×5 minutes). Cell pellets were resuspended in 0.5 mL of FACS buffer/well (∼10^6^ cells/mL) and transferred to 12×75 mm Falcon 5 mL round-bottom polystyrene FACS tubes. Labeled cells were sorted by fluorescence using a BD FACSCalibur Flow Cytometer (BD Biosciences) and a minimum of 30,000 events were recorded per sample using BD CellQuest Pro software. GFP and YOPRO-1 were detected with an FL1 detector, mRFP with FL2, and propidium iodide with FL3. Prior to each experiment, the flow cytometer was calibrated against three control groups: (1) unstained ST14A cells to gate autofluorescence, (2) unstained ST14A cells expressing GFP- or mRFP-tagged httex1-25Q alone to identify transfected cells, and (3) stained ST14A cells pre-treated with H_2_O_2_ for maximal PI or YOPRO-1 uptake. Using BD CellQuest Pro software, data were displayed in two-color dot-plot formats on a log-scale, and cell death was expressed as a percentage by dividing the number of transfected cells that stained with PI or YOPRO-1 in the upper right quadrant by the total number of transfected cells (summation of the upper and lower right quadrants). Cell death percentages were normalized to that of ST14A cells expressing non-expanded httex1-25Q. Toxicity of full-length ataxin-3 (containing either normal or expanded polyglutamine) was determined in a similar manner by FACS.

For analyzing the aggregation of httex1-72Q-GFP by FACS, an analogous protocol was employed, except that filtered cells were resuspended in FACS buffer lacking PI or YOPRO-1 and sorted on the basis of GFP intensity after calibrating against untransfected ST14A cells.

For measuring oxidative stress, filtered cells were resuspended in 1× Ca^2+^- and Mg^2+^-free PBS containing 10 µM dihydroethidium (Sigma) from a fresh stock in DMSO. Cells were stained for 20 minutes in the dark, washed with 1× Ca^2+^- and Mg^2+^-free PBS, and immediately analyzed by flow cytometry after calibrating against (1) unstained ST14A cells, (2) ST14A cells expressing non-expanded httex1-25Q-GFP or GFP-ataxin-3-24Q, and (3) DHE-stained ST14A cells to exclude steady-state oxidation levels. Ethidium bromide fluorescence was detected with an FL3 detector, and the normalized percentage of transfected cells that oxidized DHE above background was calculated similar to above.

### Statistical analysis

All values represent the mean of at least three independent experiments (±SEM). Statistical significance was determined by one-way ANOVA using Minitab or Statview statistical software. P values<0.05 were designated as statistically significant.

## Supporting Information

Figure S1 scFv-C4 fluorobody potently blocks mutant httex1 aggregation and co-localizes perfectly with soluble httex1 in live cells. ST14A cells were transiently transfected with GFP-tagged scFv-C4 fluorobody and/or mRFP-tagged httex1-72Q, and representative live-cell images were captured 48 hours post-transfection as described in Materials and Methods. In the absence of intrabody, httex1-72Q-mRFP rapidly forms cytoplasmic aggregates. Upon co-transfection with scFv-C4 fluorobody, aggregation of httex1-72Q-mRFP is potently inhibited, and scFv-C4 fluorobody co-localizes perfectly with soluble httex1 in the cytoplasm. In contrast, scFv-C4 fluorobody localizes to the nucleoplasm and cytoplasm in the absence of httex1 substrate. Neither GFP-tagged fluorobody nor mRFP-tagged httex1-72Q was detected outside of expected spectra.
(6.25 MB TIF)
