# Supplementary figures and images for: Conformational Targeting of Fibrillar Polyglutamine Proteins in Live Cells Escalates Aggregation and Cytotoxicity
Source: PLoS One. 2009 May 28;4(5):e5727. doi: 10.1371/journal.pone.0005727 (PMC2683928; doi:10.1371/journal.pone.0005727)

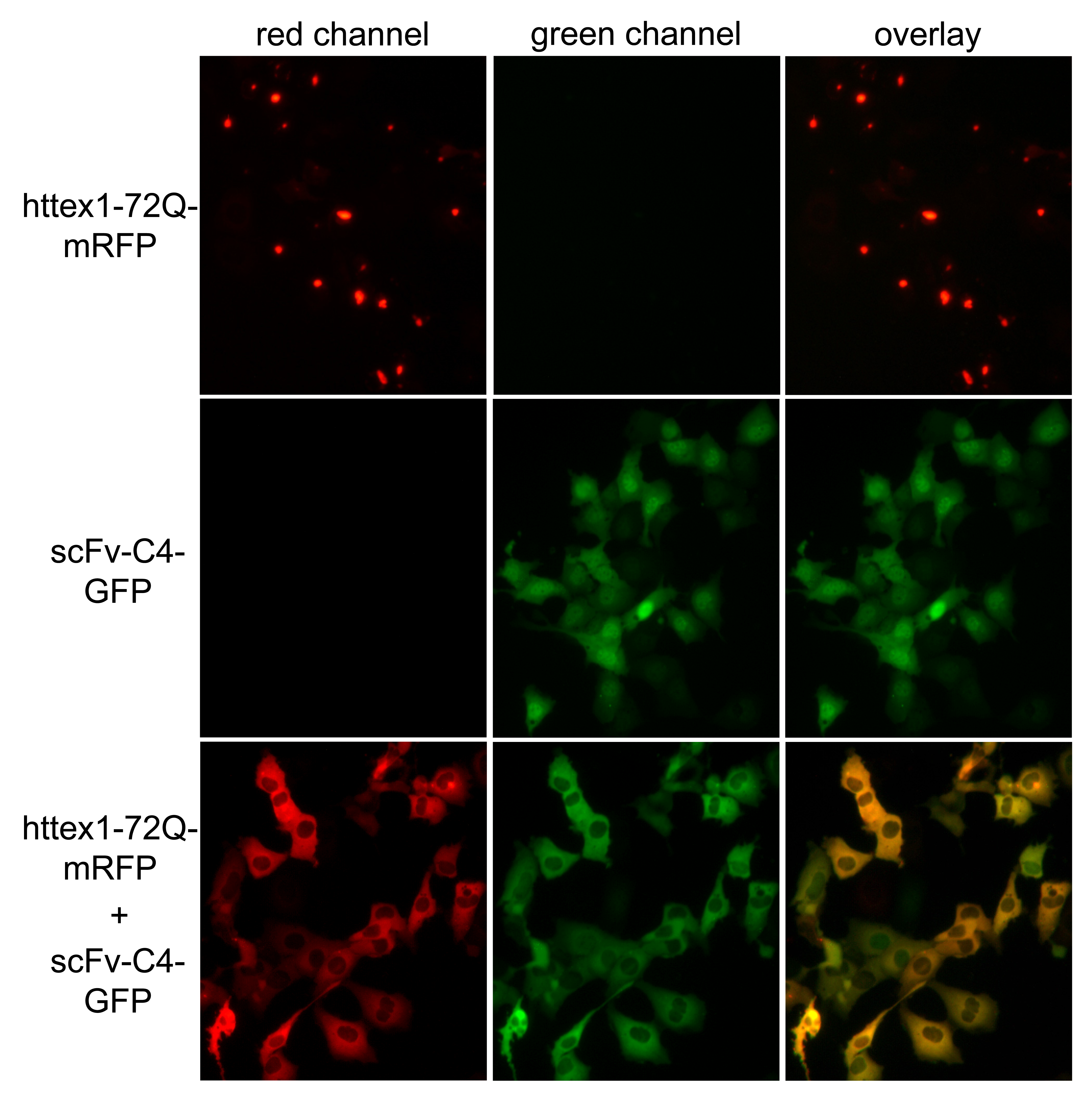

Supplement: Figure S1 — scFv-C4 fluorobody potently blocks mutant httex1 aggregation and co-localizes perfectly with soluble httex1 in live cells. ST14A cells were transiently transfected with GFP-tagged scFv-C4 fluorobody and/or mRFP-tagged httex1-72Q, and representative live-cell images were captured 48 hours post-transfection as described in Materials and Methods. In the absence of intrabody, httex1-72Q-mRFP rapidly forms cytoplasmic aggregates. Upon co-transfection with scFv-C4 fluorobody, aggregation of httex1-72Q-mRFP is potently inhibited, and scFv-C4 fluorobody co-localizes perfectly with soluble httex1 in the cytoplasm. In contrast, scFv-C4 fluorobody localizes to the nucleoplasm and cytoplasm in the absence of httex1 substrate. Neither GFP-tagged fluorobody nor mRFP-tagged httex1-72Q was detected outside of expected spectra. (6.25 MB TIF) [file pone.0005727.s001.tif]
